# Prospects for land-use sustainability on the agricultural frontier of the Brazilian Amazon

**DOI:** 10.1098/rstb.2012.0171

**Published:** 2013-06-05

**Authors:** Gillian L. Galford, Britaldo Soares-Filho, Carlos E. P. Cerri

**Affiliations:** 1 Gund Institute for Ecological Economics, University of Vermont, 617 Main Street, Burlington, VT 05405, USA; 2 Centro de Sensoriamento Remoto, Universidade Federal de Minas Gerais, Avenida Antônio Carlos 6627, Belo Horizonte, 31270-901 Minas Gerais, Brazil; 3 Departamento de Ciência do Solo, Escola Superior de Agricultura Luiz de Queiroz, Universidade de São Paulo, Avenida Paduas Dias 11, Piracicaba, São Paulo, Brazil

**Keywords:** Amazon, agriculture, greenhouse gases, rural development, soya bean

## Abstract

The Brazilian Amazon frontier shows how remarkable leadership can work towards increased agricultural productivity and environmental sustainability without new greenhouse gas emissions. This is due to initiatives among various stakeholders, including national and state government and agents, farmers, consumers, funding agencies and non-governmental organizations. Change has come both from bottom-up and top-down actions of these stakeholders, providing leadership, financing and monitoring to foster environmental sustainability and agricultural growth. Goals to reduce greenhouse gas emissions from land-cover and land-use change in Brazil are being achieved through a multi-tiered approach that includes policies to reduce deforestation and initiatives for forest restoration, as well as increased and diversified agricultural production, intensified ranching and innovations in agricultural management. Here, we address opportunities for the Brazilian Amazon in working towards low-carbon rural development and environmentally sustainable landscapes.

## 1. Greenhouse gas emissions from the Brazilian Amazon

Brazil draws global attention as a top emitter of carbon dioxide (CO_2_) from land-use change and deforestation, while simultaneously serving as custodian of the world's largest tropical forest. The Amazon forest holds about one-tenth of the global carbon in terrestrial ecosystems and an equal share of global net primary production, sequestering 0.49 ± 0.18 Pg C each year [1,2]. The Brazilian Amazon lost approximately 20 per cent of its forests between 1970 and 2011 [3], and 40 per cent of the cerrado (savannah) vegetation has been converted to agriculture [4]. Brazil is now a major producer of soya bean, vying for the spot as top global exporter with the USA. Since 1990, Brazilian soya bean production has increased by nearly 50 million tonnes, a third of which came from the Amazon state of Mato Grosso [5]. Less well known is the expanding maize industry in Brazil. In the past decade, Brazil has become one of the top five exporters of maize, with 60 per cent of this production increase coming from Mato Grosso [5,6]. These agricultural activities create new sources of greenhouse gas emissions and, while they may be lower than deforestation emissions, these emissions still need to be addressed [7]. Here, we present how agricultural emissions can be and are reduced while increasing farm production as a means of pursuing low-carbon rural development and environmentally sustainable working landscapes.

## 2. Policies for reducing deforestation

Brazil has already adopted multiple strategies to address deforestation and resulting CO_2_ emissions. First, roughly 50 per cent of the remaining Amazon forest has protected area status, including indigenous reserves, sustainable use production forests and reserves, strictly protected forests, military lands and private natural heritage reserves (see Coe *et al.* [8]). This protects a large carbon reserve and allows the country to focus on policies to reduce deforestation emissions outside the protected areas.

These policies include the United Nations Reducing Emissions from Deforestation and Degradation (REDD+), national and regional zoning or land-use planning, the Brazilian Forest Code, and the Brazilian National Policy on Climate Change. REDD+ is a mechanism of payment incentives for landowners to manage forests to store carbon. Several projects are already running including in the Juma Sustainable Development Reserve that was designed to prevent deforestation on 366 000 hectares of forest with an estimated carbon offset of 210 million tonnes of CO_2_ by 2050 [9,10]. Second, national and sub-national zoning and land-use planning, such as the Legal Amazon Ecological–Economic Macro-Zoning (MarcoZEE), is meant to promote low-carbon rural development by maximizing conservation and economic production as tailored to the region. Next, the Brazilian Forest Code governs forest conservation on private property although there are opportunities for increased protection in the cerrado. Finally, the Brazilian National Policy on Climate Change establishes the goals of reducing forest and cerrado deforestation levels by 80 per cent and 40 per cent, respectively, relative to the 1996–2005 baseline. All of these policies are discussed in more detail by Coe *et al*. [8].

It should be noted that some of these policies are difficult to apply to lands without clear land tenure; decreasing demands for new clearing may require that land tenure issues be resolved through a comprehensive land titling and zoning programme [11]. Minus that caveat, statistical analysis of controls on deforestation shows that conservation policies, particularly with effective enforcement, can decrease deforestation in the Amazon and therefore reduce carbon emissions [12]. Thus, we focus on the other source of Amazon emissions, agriculture, for the remainder of the paper.

## 3. Greenhouse gas emissions from agriculture

Farmers in Mato Grosso, the heartland of Amazon agriculture, are rapidly adopting double-cropping schemes to grow soya beans and maize as two separate harvests on the same field within a single rainy season. Since 2005, double cropping has shifted from 35 per cent of the state's cropland areas to 65 per cent [13], tripling Mato Grosso's maize production [5]. The growth of these croplands is likely to continue and may result in mixed impacts on the environment, including emissions of the greenhouse gas nitrous oxide (N_2_O) from nitrogen fertilizer or CO_2_ from soil tillage, although these emissions are likely to be small compared with deforestation emissions [14].

Curtailing losses of soil carbon and nitrogen means increased soil stocks and reduced greenhouse gas emissions, benefitting both the farmer and the global environment. Soil organic carbon is an important component for water retention, enhancing soil biodiversity and absorption of nutrients that might otherwise be leeched. Crop growth is enhanced by improving nitrogen use efficiency largely by decreasing losses of N, such as N_2_O, by following the four ‘Rs’: carefully applying the right nutrient source, at the right rate, at the right times and in the right place [15]. Unfortunately, the high rates of return to agriculture increase the opportunity costs of conservation [16] as well as the costs of enforcement [17], and increase pressure on the Brazilian government to soften environmental laws, such as the Brazilian Forest Code, or protected areas^1^ [18] for agriculture, resulting in increased emissions from deforestation. In response, new incentives for sustainable production have minimized new deforestation for agricultural croplands and pastures, as discussed in §§5 and 6.

## 4. Agricultural opportunities for reducing emissions

There are many opportunities for reductions in greenhouse gas emissions from Amazon agriculture, including conservation agriculture and associated practices, rehabilitation of pastures, and new systems of integrated production. We discuss each of these opportunities and their impacts on greenhouse gas emissions in this section.

Brazilian farmers have already demonstrated how rapidly, and widely, improved management techniques can be adopted. Conservation agriculture (CA) management techniques maintain vegetation cover, dead or alive, on soil, avoid ploughing or tilling the soil and encourage crop rotation, including cover crops. CA began in the USA in the 1930s and took off in earnest in the 1960s, yet today the practice is in use in only 41 per cent of croplands [19]. Brazil has demonstrated a rapid adoption of CA as it spread from 0 to 53 per cent of all cropland area from 1980 to 2006 [20]. CA increases soil carbon and nitrogen content, thereby also increasing the soil's water-holding capacity as well as nutrient and water-use efficiencies of the crops. Additional benefits to the agro-ecosystem include decreased run-off, decreased erosion and improved water quality. The impacts of CA on greenhouse gas emissions in the Amazon are not thoroughly documented. In the one study in Mato Grosso, Carvalho *et al.* [21] found that the conversion of a conventional tillage field to CA management increased soil carbon sequestration by 0.38 Mg ha^–1^ yr^−1^. This study also found the highest N_2_O emissions coming from CA, indicating a potential trade-off between increased carbon storage and nitrogen emissions. After accounting for emissions of N_2_O and CH_4_, they found a net C sequestration increase of 0.23 Mg C ha^−1^ yr^−1^ when converting from conventional to CA [21]. The largest benefit from CA on greenhouse gas emissions is the reduction of about 60 per cent in fossil fuel (diesel) consumption owing to the reduced use of machinery.

Even when practised singularly, CA practices can have co-benefits to the ecosystems and agriculture. Intensified row-crop agriculture can sequester carbon in soils if managed under no-tillage practices, as practised in most of the Amazon. For example, no-tillage management in cerrado areas increased soil organic carbon storage by a factor of 1.08 ± 0.06 (approx. 8%) relative to stocks under native conditions. More modest increases (1.01 ± 0.17) in soil organic carbon have been documented in cerradão (tall, dense savannah woodland) and Amazon forest conditions [22]. In Rondônia state, southwestern Amazon, annual soil organic carbon accumulated at a rate of 0.38 Mg C ha^−1^ yr^−1^ when conventionally tilled rice was converted to soya bean under no-tillage [21]. In parts of the southern Amazon, farmers are using cover crops to improve soil conditions, such as: (i) ‘pé de galinha’ (*Chloris gayana*) or ‘nabo forrageiro’ (*Raphanus sativus* L.), which are deep-rooting grass and radish plants, respectively, that can break up soil aggregates in no-till systems, (ii) millet incorporation back into the soils to build soil organic carbon stocks, and (iii) sorghum use during the dry season for cattle grazing.

Another new practice in the Amazon is the rehabilitation of degraded agricultural lands. Rehabilitation typically takes underused or poorly managed lands, and uses a combination of techniques to restore productivity. Cerri *et al.* [23] found that fertilization of degraded pastures was one of the most effective ways to restore productivity. While this may lead to increased greenhouse gas emissions (N_2_O) compared with the degraded pasture, it may be advantageous compared with the emissions associated with deforestation to create new pasture lands. Additionally, more productive pastures will increase soil carbon storage, serving as a carbon sink [23].

The newest innovations in Amazon agriculture have yet to be studied for their greenhouse gas impacts. For example, ranchers-turned-farmers are bringing cattle back through a system called ‘integration’ that has been heavily promoted by the Brazilian agricultural agency, EMBRAPA, in central Mato Grosso since the mid-2000s. Integration agriculture is rapidly evolving and involves rotating soya beans with forage crops to fatten cattle during the dry season. Once the rains come, another cash crop is planted, sometimes returning to soya beans or cycling through other crops such as cotton. The dry season forage crops could be sorghum or millet or a nutrient-rich pasture grass such as Tanzania (*Panicum maximum* cv. *Tanzania*) that are grazed at higher stocking rates than typical pastures. Grazing 6 AU ha^−1^, instead of the more typical 0.5–1 AU ha^−1^, is acceptable as pasture degradation is not a concern because of the rapid conversion back to crops. Questions remain regarding the biogeochemical impacts of integration systems. Will grazing increase soil organic matter or cause compaction of the soils? Will methane emissions increase from the high stocking rates and is that offset by carbon transfer to soils? What are the nitrogen or other fertilizer requirements for the integration systems to have sustained productivity?

## 5. Incentives to reduce agricultural emissions

Practices that improve farm productivity and the local environment are not always obvious choices and may require special training or the help of extension services to increase adoption. EMBRAPA has been highly effective in networking and advising farmers, increasing the number of *municipios* (counties) receiving advice from 10 per cent in the 1960s to more than 70 per cent by the 1980s, and still maintains high levels today [24]. Farmers are responsive to environmental concerns when provided with enough information to act locally and economically. EMBRAPA has been instrumental in helping farmers adopt conservation agriculture, rehabilitate lands and experiment with integration practices.

In addition to extension services from EMBRAPA, cattle ranchers and soya farmers may find incentives for achieving voluntary emissions reductions through registries for responsible land management [17] and through the perception among farmers that standing forests will soon gain value through a carbon market [16]. Voluntary initiatives and perceptions have been bolstered by international commodity certification systems as well as by moratoria on growing soya and beef on recently cleared lands [25]. Registry programmes of social–environmental responsibility for landowners, such as the one developed by Aliança da Terra, have been successful in the Xingu headwaters of Mato Grosso state. This registry works with producers and other social and scientific partners to identify, recognize and reward sustainable producers with the goal of working with more than 20 million ha by the year 2017. Such initiatives will be key to promoting best land-use practices [26] and also to supplying international markets demanding environmentally sound products such as deforestation-free soya. In another example, after 2014, Dutch companies will only buy certified soya with the standards set by the Round Table on Responsible Soy [27]. The consolidation of these economic and political factors to influence markets, laws and regulation will promote the viability and attractiveness to farmers of using environmentally sound management.

## 6. Policy mechanisms to reduce agricultural emissions

The cornerstone of Brazil's National Climate Change Plan is decreased greenhouse gas emissions through reduced pressures on the forest margins. This is to be achieved through the intensification of the cattle industry in order to spare land for soya bean and sugarcane production [28]. Both Brazil's National Climate Change Plan and the proposed Nationally Appropriate Mitigation Action aim to constrain new deforestation through intensified cattle production. To reach this goal, federal credit programmes, as well as research activities, are aligning to support intensification. However, there is no guarantee of decreased deforestation and the enforcement of existing environmental laws remains inconsistent in the frontier.

An example of government-supported low-carbon agricultural development is Programa ABC, which stands for Agriculture de Baixo Carbono, or low-carbon emission agriculture [18,29]. One of the objectives of this programme is to rehabilitate degraded pastures. The goal is to improve about 15 million ha in the next decade that would lead to a reduction in emissions from 83 to 104 million tonnes of CO_2_ equivalent as compared with extensified production. In addition, Programa ABC encourages an extra 8 million ha that would avoid 16–20 million tonnes of CO_2_ equivalent in emissions compared with current practices. Integrating crops, livestock and forestry is another line of action that the government aims to develop on an additional 4 million ha. Also planned are the capture and use of methane gas produced by animals that would otherwise be a direct emission to the atmosphere (avoiding about 7 million tonnes of CO_2_ equivalent), the use of biological nitrogen fixation and expansion of commercial forestry from 6 to 9 million ha.

## 7. Outlook

Are these outcomes plausible? Can the Amazon frontier sustain continued agricultural growth without increasing emissions or pressures on deforestation? Gouvello *et al.* [28] examined land-cover and land-use change and forestry scenarios to simulate the impacts of future agricultural expansion in Brazil. In the low-carbon scenario, cropland for grains in Mato Grosso would expand by 20 per cent, from 7.3 to 8.8 million ha. This scenario assumes that Brazil will replace 80 per cent of its gasoline consumption with ethanol and meet 10 per cent of the global ethanol demand by 2030. The scenario also assumes an expansion of commercial forest plantations to eliminate deforestation for charcoal production by 2017 and an offset of 46 per cent of coal used in iron and steel production by 2030. In addition, an effort to restore 44 million ha of forest would take place. In Mato Grosso, the low-carbon model demonstrates that it is possible to free up pasture for cropland by increasing livestock productivity at the same time that deforestation is reduced by 95 per cent by 2030. Macedo *et al.* [30] demonstrated the plausibility of this scenario showing that increased agricultural production in Mato Grosso between 2006 and 2012 has come at minor costs to the region's forests and without leakages to nearby states. Therefore, the low-carbon scenario may be achievable.

## 8. Conclusions

The agricultural frontier of the Brazilian Amazon demonstrates innovation and advancement of agricultural production and environmental protection. This region can be a global leader and an archetype of environmental sustainability in working agricultural landscapes. Brazil has used both top-down and bottom-up approaches to reducing deforestation and has coupled these with excellent scientific research and outreach programmes for landowners. Such a multi-tiered approach must also be used to minimize emissions of greenhouse gases from the agricultural sector. To this end, it will be crucial that intensification programmes are associated with complementary forest restoration initiatives. Many of the practices and programmes discussed here can be applied at any farm scale; however, some of the larger financial incentives or policies may not be accessible or applicable to small farmers unless they are directly targeted. Another caution is that agricultural production intensification practices may introduce new environmental concerns, such as increased use of pesticides. Not addressed in this paper are the additional emissions associated with transporting, processing and consuming agricultural process. Complete life cycle analysis approaches are developing in Brazil that will allow us to account for all emissions along the commodity chain [31]. With the perspective of avoiding unintended environmental problems that have resulted in other parts of the world, Brazil can embrace and execute environmentally sustainable agriculture through the twenty-first century. It is clear that agricultural development will and should continue; as such, it is crucial to embrace sustainable practices and maintain low deforestation rates. Mato Grosso is already leading the way by reducing emissions through voluntary social–environmental responsibility programmes and certification programmes. Further research and promotion of increased nitrogen use efficiency to minimize greenhouse gas emissions and increase farm profits, as well as initiatives aimed at reducing the costs of forest code compliance, are the next steps to ensuring low-carbon rural development.

## Endnote

1 Medida Provisória No. 542 de 12 de Agosto de 2011, 2011.
